# Transient Myeloproliferative Disorder (TMD), Acute Lymphoblastic Leukemia (ALL), and Juvenile Myelomonocytic Leukemia (JMML) in a Child with Noonan Syndrome: Sequential Occurrence, Single Center Experience, and Review of the Literature

**DOI:** 10.3390/genes15091191

**Published:** 2024-09-10

**Authors:** Marta Arrabito, Nicolò Li Volsi, Manuela La Rosa, Piera Samperi, Giulio Pulvirenti, Emanuela Cannata, Giovanna Russo, Andrea Di Cataldo, Luca Lo Nigro

**Affiliations:** 1 Center of Pediatric Hematology Oncology, Azienda Policlinico di Catania, 95100 Catania, Italy; marta-arrabito@hotmail.it (M.A.); n.livolsi@outlook.it (N.L.V.); psamperi@unict.it (P.S.); e.cannata80@gmail.com (E.C.); diberuss@unict.it (G.R.); adicata@unict.it (A.D.C.); 2 Department of Clinical and Experimental Medicine, University of Catania, 95100 Catania, Italy; 3 School of Medical Genetics, University of Catania, 95100 Catania, Italy; 4 Cytogenetic-Cytofluorimetric-Molecular Biology Lab, Center of Pediatric Hematology Oncology, Azienda Policlinico di Catania, Via Santa Sofia 78, 95123 Catania, Italy; larosa_manuela@libero.it; 5 School of Pediatrics, University of Catania, 95100 Catania, Italy; giulpulv@hotmail.it

**Keywords:** Noonan syndrome, children, PTPN11 mutation, acute lymphoblastic leukemia, juvenile myelomonocytic leukemia, hematological diseases

## Abstract

Noonan syndrome (NS) is an autosomal dominant disorder that varies in severity and can involve multiple organ systems. In approximately 50% of cases, it is caused by missense mutations in the *PTPN11* gene (12q24.13). NS is associated with a higher risk of cancer occurrence, specifically hematological disorders. Here, we report a case of a child who was diagnosed at birth with a transient myeloproliferative disorder (TMD). After two years, the child developed hyperdiploid B-cell precursor acute lymphoblastic leukemia (BCP-ALL), receiving a two-year course of treatment. During her continuous complete remission (CCR), a heterozygous germline mutation in the *PTPN11 gene* [c.218 C>T (p.Thr73lle)] was identified. At the age of ten, the child presented with massive splenomegaly, hyperleukocytosis, and thrombocytopenia, resulting in the diagnosis of juvenile myelomonocytic leukemia (JMML). After an initial response to antimetabolite therapy (6-mercaptopurine), she underwent haploidentical hematopoietic stem cell transplantation (HSCT) and is currently in complete remission. The goal of this review is to gain insight into the various hematological diseases associated with NS, starting from our unique case.

## 1. Introduction

**Figure 1:**
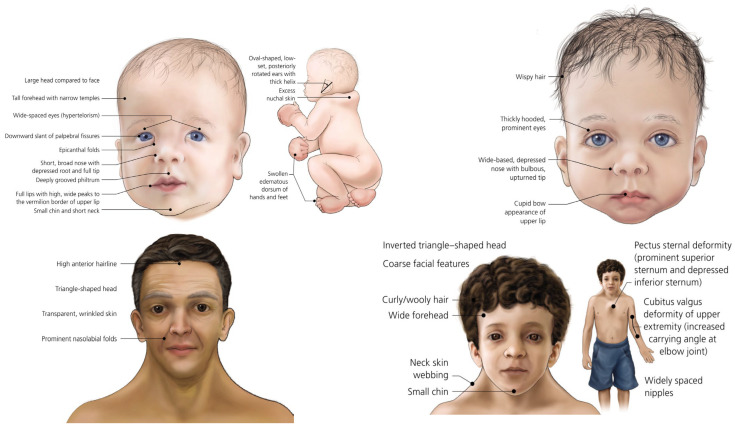
Phenotypical features of Noonan syndrome, adapted from Ref. [13]. Public domain images of Noonan syndrome, from the National Human Genome Research Institute, National Institute of Health, Bethesda, Maryland, USA.

Noonan syndrome (NS) is a genetic disorder that varies in severity and can involve multiple organ systems over the patient’s entire lifetime. To date, an estimated 1:1000–1:2500 individuals have been identified with NS [1]. This syndrome is characterized by distinctive facial features, e.g., a high forehead; widely spaced eyes with scaled palpebral fissures; low-set and posteriorly rotated ears; short stature; congenital heart disease, such as pulmonary valve stenosis; and delayed psychomotor development (Figure 1). Furthermore, these patients bear a very high tumor-risk during childhood. Indeed, NS is mostly caused by pathogenic variants in the *PTPN11* gene that lead to a threefold increase in the risk of malignancy [2]. The principal types of malignancies associated with NS are acute lymphoblastic leukemia (ALL) [3] and solid tumors (glioma, rhabdomyosarcoma, lymphoma) [4]. The diagnosis of NS is established in a proband with suggestive clinical signs and heterozygous pathogenic variations in *BRAF*, *KRAS*, *MAP2K1*, *MRAS*, *NRAS*, *PTPN11*, *RAF1*, *RASA2*, *RIT1*, *RRAS2*, *SOS1*, or *SOS2* [1] or a heterozygous variant or variants of biallelic pathogens in *LZTR1* identified by molecular genetic testing [5]. Several additional genes associated with an NS phenotype have been identified in fewer than ten individuals. NS is often inherited in an autosomal dominant manner. While many individuals with autosomal dominant NS exhibit a de novo pathogenic variant, an affected parent is recognized in 30% to 40% of families [1]. Prenatal testing and preimplantation genetic testing are possible, especially when NS-related pathogenic variants have been identified in an affected family member [5]. Regarding the proportions attributed to specific pathogenetic variants, the *PTPN11* gene is involved in 50% of cases [6], followed by *SOS1* (10–13%) [7]; *LZTR1* (~8%) [6]; *RAF1-RIT1* (5%) [8]; *KRAS* (<5%) [9]; *SOS2* (~4%) [10]; *BRAF-MAP2K1* (<2%) [11]; *MRAS-NRAS-RRAS* (<1%) [12]. Molecular genetic testing approaches may include a combination of gene-targeted testing (multigene panel) and comprehensive genomic testing (exome sequencing or genome sequencing), depending on the phenotype. Individuals with phenotypic features suggestive of NS can be diagnosed using gene-targeted testing. Sometimes, the genetic identification of NS is performed after the diagnosis of the related hematological disorders, as in our case. Ultimately, the goal of this review is to clinically and genetically analyze the various hematological diseases associated with NS, starting with the description of a peculiar case diagnosed in our center. Moreover, based on this single case, we performed a retrospective analysis in order to identify the “real” incidence of NS among children with ALL.

## 2. Materials and Methods

For the identification of *PTPN11* mutations among the children affected by hematological malignancies cited in this manuscript, a next generation sequence (NGS) technology was applied, followed by a Sanger sequencing analysis as the confirmation test. Selection criteria were based only on clinical evaluation (Figure 1).

## 3. Biological Mechanisms Shared between NS and Hematological Malignancies

In 2001, *PTPN11*, encoding *SHP2*, a non-receptor protein tyrosine phosphatase playing a relevant role in intracellular signaling and several developmental processes, was identified as the major Noonan syndrome (NS, MIM: PS163950) disease gene using a positional candidacy approach [14]. In 2003, missense mutations in *PTPN11* were discovered as somatic events occurring in juvenile myelomonocytic leukemia (JMML), as well as in childhood myelodysplastic syndromes (MDS) and acute myeloid leukemia (AML) [15], marking a milestone in the development of the understanding of the underlying mechanisms of these hematological malignancies. The main pathway involved in this association is the RAS-MAPK pathway. This signaling cascade flows through the pathway, together with the proteins positively and negatively controlling the cascade; signaling upregulation in RASopathies like NS results from enhanced activity of RAS proteins (i.e., HRAS, KRAS, NRAS, MRAS, RRAS, RRAS2, and RIT1), upstream positive signal transducers and regulators (i.e., SHP2, SOS1, and SOS2), proteins favoring transmission of RAS signaling to downstream transducers (i.e., MRAS, SHOC2, and PPP1CB), and tiers of the MAPK cascade (i.e., BRAF, RAF1, MAP2K1, MAP2K2, and MAPK1) [16]. Signaling upregulation also results from inefficient signaling switch-off operated by multiple feedback mechanisms (i.e., defective/impaired function of CBL, neurofibromin, LZTR1, SPRED1, and SPRED2), leading to an uncontrolled proliferation [16]. All the above listed genes are involved in generating MDS, JMML, AML, and hyperdiploid (HeH) ALL [17].

## 4. Results (Cases from Center of Pediatric Hematology Oncology in Catania—Italy)

### 4.1. Case 1

**Figure 2:**
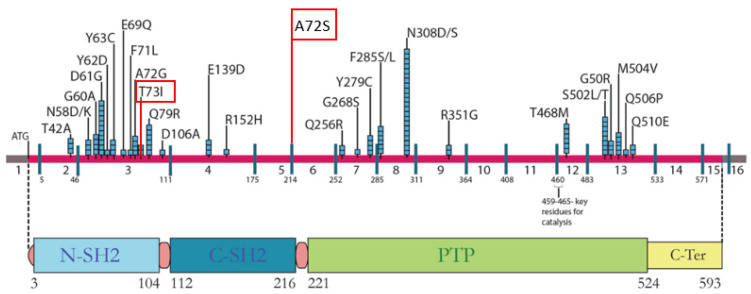
Location of PTPN11 pathogenic variants shown, along with exons and SHP-2 functional protein domains [20]. The red variants represent two of the three mutations detected in our patients (Cases 1 and 2, respectively).

A female child (UPN 1045685) presented at birth with an angiomatous ulcerated neoformation in the right arm. After the first days of life, she was admitted to the neonatal intensive care unit due to respiratory distress and sepsis, caused by Pseudomonas aeruginosa. During the hospitalization, a skin biopsy of the right arm lesion was performed, and diagnosis of juvenile xantogranuloma-like Langherans histiocytosis was made (Table 1). She was also diagnosed with Wolf–Parkinson–White Syndrome. After one month, because of the presence of leukocytosis, thrombocytopenia, and splenomegaly, she was diagnosed with transient myeloproliferative disorder (TMD), juvenile myelomonocytic leukemia (JMML)-like, which disappeared in a few months without any treatment: the number of CD34^pos^ immature elements (20%) was slightly declined, with a spontaneous increase in platelets (Table 1). At the age of two years, the patient was diagnosed with acute lymphoblastic leukemia (ALL), with common immunophenotype, lack of translocations, and a DNA index > 1 (with hyperdiploidy—HeH ALL) (Table 1). Therefore, the child was enrolled in the AIEOP-BFM ALL 2009 protocol [18]. She presented with a good clinical outcome, showing high sensitivity to conventional treatment and no particular side effects or adverse events. She was classified as a prednisone good responder (PGR) and as bone marrow (BM) compatible, with standard risk (SR), at day+15. The detection of minimal residual disease (MRD) during induction indicated that the patient should be assigned to the intermediate risk group (MR). She also received the experimental arm treatment, including nine additional administrations of PEG-asparaginase (Table 1). At the age of seven, the patient’s phenotypic features became clearer, a genetic examination was performed (Table 1), and a diagnosis of Noonan syndrome was made (c.218 C>T p.Thr73lle mutation in the PTPN11 gene in heterozygotes state) (see Figure 2). At the age of ten years, she presented with an increased abdominal circumference associated with abdominal pains. An ultrasound examination was performed, revealing a marked hepatosplenomegaly (LD 190 mm) and mild peritoneal effusion in perisplenic and perihepatic region. The blood count showed leukocytosis (WBC 64,000/mmc) and thrombocytopenia (PLT 24,000/mmc). A cytofluorimetric analysis of the peripheral blood presented an increase in the myeloid immature cells (9%). Therefore, a diagnosis of JMML [hyperleukocytosis, presence of monocytes, elevated levels of Fetal Hemoglobin (Hb-F)], characterized by splenomegaly and thrombocytopenia, was made (Table 1) [1]. The patient was treated with 6-mercaptopurine (starting dose: 25 mg/die) recommending her for an allogeneic hematopoietic stem cell transplantation (HSCT), considered the treatment of choice in children with JMML. Two months later, a follow-up BM evaluation showed a decrease in the percentage of the blast cells, but after this initial response to therapy, a recurrence of leukocytosis associated with thrombocytopenia and splenomegaly occurred. A cytofluorimetric analysis of the peripheral blood showed the presence of 10% immature cells (WBC 22,850/mmc). Therefore, due to the lack of an HLA-compatible donor, even using the international registry, she underwent an haploidentical stem cells transplantation (Haplo-SCT) from her father, according to the Baltimore protocol (Table 1) [19]. Now, the patient is twelve years old and is in good clinical condition following monthly hematologic controls, with no signs of any hematological disease. Based on this case experience, we performed a retrospective analysis of 300 cases with ALL diagnosed using three consecutive protocols, identifying two additional cases with NS and ALL.

### 4.2. Case 2 (UPN 1021127)

As in the previous case, the patient received the diagnosis of NS after the occurrence of ALL. At 6 years old, she underwent surgery for pulmonary valve stenosis. Six years later, she received a diagnosis of BCP-ALL, presenting with vertebral osteolysis. She displayed the following ALL biological features: common immunophenotype, lack of translocations, and a DNA index > 1 (HeH ALL). Therefore, the child was enrolled in the AIEOP-BFM ALL 2000 protocol. Due to the presence of mild cardiomyopathy, a dose reduction of anthracyclines during the induction and re-induction phases was administered. At the end of induction, the detection of MRD indicated that she be assigned to the intermediate risk (MR) group. The treatment was completed after two years, without any particular side effects. She is currently in good clinical conditions, so far. At 14 years old, due to the detection of specific phenotypic features, such as the cardiopathy, a genetic assessment was performed, and a diagnosis of NS was determined by identifying a new *PTPN11* mutation (c.[214G>T]-(p.[Ala72Ser]) (see Figure 2).

### 4.3. Case 3 (UPN 1052079)

Unlike the other two cases, this child received the diagnosis of NS during the first months of life (germline *PTPN11* mutation), before developing ALL, because of the presence of typical facial features and pulmonary valve stenosis. At two years old, a diagnosis of B-cell precursor (BCP)-ALL was made. At the onset, we surprisingly found the same biological features as those noted in the previous cases: in particular, cytogenetic analysis showed a hyperdiploid karyotype. The child was enrolled in the AIEOP-BFM ALL 2009 protocol [18]. He showed a good prednisone response after 8 days of treatment, and the evaluation of BM at day + 15 was compatible with SR. At day + 33, a complete remission (CR) was reached, but during the induction–consolidation phase, a severe adverse event occurred, and the patient died from a Staphylococcus-related sepsis.

This rate of occurrence could identify NS as the second most common genetic disease, after Down syndrome, associated with a high predisposition to BCP-ALL. Furthermore, the presence of hyperdiploidy in all three reported cases highlighted the strong association between the expression of a hyperdiploid karyotype and the genetic aberration in the *PTPN11* gene, which has been detected in non-NS cases with somatic *PTPN11*-mutated ALL.

## 5. Discussion

Noonan syndrome (NS) could be associated with many diseases, including both hematological disorders and/or solid tumors, sharing the same mutation or a different one. Some interesting cases or series developed in the context of NS, as noted in the literature, are reported here.

### 5.1. Noonan Syndrome and Transient Myeloproliferative Disorder (TMD)

The most common hematopoietic disease in patients with NS is TMD, which is mainly diagnosed in the neonatal period or early infancy (Table 2) and is found in up to 10% of all NS cases, as reported by Niemeyer et al. [21]. Even though this is considered a benign disorder, some children could later develop JMML. Based on an unpublished observational study by the European Working Group of Myelodysplasia (MDS) and Severe Aplastic Anemia (SAA) in children (EWOG-MDS/SAA), up to 30% of children with NS and severe TMD die because of myeloid-proliferation associated with their other clinical problems. Almost all patients with NS and TMD show mutations in the *PTPN11* gene [15]. This mutation causes a gain-of-function (gof) effect. Since 2002, cases of transient abnormal myelopoiesis have been described. Ferraris et al. reported on the case of a baby diagnosed with NS based on clinical and echocardiographic characteristics and who presented hepatosplenomegaly, hyperleukocytosis, and anemia at the age of 2 months [22]. Thus, a bone marrow aspirate was performed that showed myelomonocytic hyperplasia, hyper-eosinophilia, and maturation dysplasias, excluding a malignant hematological disease. No translocations or chromosomal alterations were found. Two months later, the cell counts and hepatosplenomegaly returned to the normal range [22]. Nemcikova et al. reported on the case of a patient with NS and TMD harboring a novel heterozygous mutation of the RIT1 gene. At 7 months of age, the child was diagnosed with an iron deficiency anemia based on pallor, leukopenia, and neutropenia. He was treated with martial therapy and recovered from the anemia. After 3 months, following a severe viral pulmonary infection complicated by bacterial and fungal superinfection, leukopenia with thrombocytopenia were still evident, along with hepatosplenomegaly. The morphology of the bone marrow aspirate showed myeloid hyperplasia (42.8%) associated with the presence of 4.8% blasts and 6.8% monocytes, suggesting a myeloproliferative syndrome. The cytogenetic analysis reported a normal [46,XY] karyotype. Following the administration of steroid therapy and after several weeks, the hepatosplenomegaly and myeloproliferative syndrome resolved. Furthermore, due to facial dysmorphism, a genetic disorder was suspected, and a molecular genetic test for the causal genes of RASopathies (PTPN11, SOS1, RAF1, RIT1, HRAS, KRAS, NRAS, BRAF, MAP2K1, MAP2K2, CBL, RRAS, and SHOC2) was performed. The analysis showed missense substitution in heterozygous c.69A>T in exon 2 of the RIT1 (NM_006912.5) gene, with consequent amino acid change p.Lys23Asn, but this variant has not been found in his parents [23]. In 2011, Bastida et al. reported two cases of NS and TMD. In the first case, a three-month-old infant, diagnosed with NS at birth based on facial dysmorphisms, was described [24]. An analysis of the peripheral blood detected 10.8 g/dL of hemoglobin, leukocytosis with monocytosis, and 10% fetal hemoglobin (HbF) associated with hepatosplenomegaly. A molecular analysis identified a de novo missense mutation (F285S) in exon 8 of the *PTPN11* gene. The child underwent periodic hematological follow-up, and his blood cell count gradually returned to the normal range after 24 months [24]. The second case concerns a baby diagnosed at birth with NS due to facial dysmorphism, who at 22 days of age exhibited hyperleukocytosis with 11% monocytes, a normal platelet count, and normal hemoglobin values but a percentage of HbF close to 98%. The bone marrow analysis showed 10% mature cells and 15% monocytes. Molecular analysis revealed a mutation (D61G) in exon 3 of the *PTPN11* gene. After 2 years of follow-up, he showed normal cell counts [24]. In a paper published in 2017, O’Halloran et al. described a patient with transient JMML and NS with a *PTPN11* mutation who subsequently developed a monosomy of chromosome 7 [25]. At birth, this baby presented with dysmorphic features suggestive of RASopathy and associated with splenomegaly. Thus, microarrays were performed, revealing a heterozygous germline missense mutation in exon 13 of *PTPN11* Ser502Leu, confirming the diagnosis of NS. During the first days of life, she showed 16,300/mmc leukocytes, 126,000/mmc platelets, hemoglobin levels of 15.2 g/dL, and 10–11% myeloblasts. Conventional karyotyping and FISH revealed monosomy 7 in the peripheral blood; however, buccal swabs and skin biopsies did not detect monosomy 7, suggesting a somatic subclonal lesion limited to the hematopoietic compartment [25]. A bone marrow biopsy at 6 weeks of age showed trilineage dysplasia, with 9% myeloblasts and peripheral leukocytosis with absolute monocytosis. A cytogenetic analysis showed monosomy 7 in 74% of cells. Another bone marrow evaluation was performed at 5 months of age, revealing hypercellularity with similar morphology and 7% blasts. Given the persistence of splenomegaly, absolute monocyte counts of 2280/mmc, 9% blasts, *PTPN11* mutation, monosomy 7, circulating myeloid precursors, and WBC 16,300/mmc, a diagnosis of JMML was made, and bone marrow transplant was considered as the treatment of choice. However, a spontaneous resolution of JMML occurred. Therefore, it was decided to follow the patient with a wait-and-watch strategy, performing blood tests every 4 months and annual bone marrow biopsies. Although the criteria for JMML were unmet, the monosomy of chromosome 7 persisted [25].

### 5.2. Noonan Syndrome (NS) and Juvenile Myelomonocytic Leukemia (JMML)

JMML is a rare and aggressive myelodysplastic neoplasm of early childhood, associated with excessive monocytic and macrophage proliferation. Subjects affected by JMML present splenomegaly, monocytosis, anemia, thrombocytopenia, and an elevated fetal hemoglobin (HbF) rate. In patients with NS, there is a high risk of developing several types of childhood cancers, including JMML [26] (Table 3). Roughly, 90% of children with JMML show mutations in one of the following five genes: *PTPN11*, *NRAS*, *KRAS*, *NF1*, or *CBL*. Because of the size and complexity of the *NF1* gene, the diagnosis of NF1 in children with JMML was previously based on the presence of six or more café-au-lait spots and an affected parent. A heterozygous mutation can occur in the *PTPN11* and *RAS* genes, either at the somatic or germline level. Consequently, genetic screenings of leukemic cells have to be followed by studies in non-hematopoietic tissue such as fibroblasts, nail cells, hair bulbs, or buccal epithelial cells [21]. In general, in NS, JMML exhibits a more benign course. The associated variants are different from the somatic pathogenic variants in *PTPN11*-associated JMML, which, when present as germline variants, are associated with neonatal-lethal NS [27]. In 1999, Choong et al. described the overall life condition of an infant, born at the 28th week, who presented at birth with phenotypic characteristics attributable to NS. Furthermore, at birth splenomegaly, thrombocytopenia, and leukocytosis (with increased percentage of monocytes) were observed. A bone marrow analysis was then performed, establishing the diagnosis of JMML. The baby suffered from chronic respiratory infections, which led to death at the age of four months [28]. Cheong et al. described an infant patient, born at 36 weeks of gestation, who presented clinical characteristics compatible with a diagnosis of NS at birth. At approximately 1 week of age, he presented hepatosplenomegaly with anemia, thrombocytopenia, leukocytosis (with monocytosis), and 2–6% blasts. JMML was demonstrated in a subsequent bone marrow aspirate. A molecular analysis showed a *PTPN11* mutation, with a C-T substitution at nucleotide 218. Despite the effort to arrange a bone marrow transplantation, the patient died at the age of 10 weeks due to respiratory failure [29]. Ortiz et al. also described a case of JMML in NS featuring an alteration at the *KRAS2* gene level. The study was conducted on an adolescent (16 years old) with a history of splenomegaly, lymphadenopathy, short stature, and delayed sexual development, presenting with abdominal distension, orthopnea, and night sweats. Upon careful analysis, it was observed that he displayed numerous characteristics that clinically suggested NS. In terms of blood tests, he showed a normal leukocyte count, with monocytosis, anemia, and thrombocytopenia; the bone marrow aspirate showed marked hypercellularity and monocytes between 5 to 6%, with myeloblast levels at less than 2%. A splenectomy was then performed. The patient subsequently died. Following the patient’s death, a genetic analysis showed a missense mutation in codon 13 of the *KRAS2* gene, confirming the diagnosis of NS and JMML [30]. Furthermore, a retrospective project, conducted by Strullu M. et al. in 2014, analyzed a group of 1550 patients diagnosed with NS; 641 of them (41%) revealed a *PTPN11* mutation. Among these 641 patients, 621 did not meet the criteria for a diagnosis of JMML; however, 16 of these presented myeloproliferative characteristics (605 did not), and 20 out of 621 manifested the criteria for a diagnosis of JMML in NS [26].

### 5.3. Noonan Syndrome (NS) and Acute Lymphoblastic Leukemia (ALL)

As previously mentioned, a germline mutation of the *PTPN11* gene has been detected in the development of almost 50% of NS cases [14]. This gene encodes for SHP-2, a phosphatase protein that is involved in the regulation of the intracellular signaling activity and which is required for the activation of the RAS/MAPK cascade. The over-activation of SHP-2 due to a mutation in the *PTPN11* gene has been shown to be associated with some hematological malignancies such as B-cell precursor (BCP) ALL as a somatic aberration in 7% of cases [31]. Thus, *PTPN11* mutations are not only associated with the occurrence of NS as germline aberrations, but they also play an important role in the development of ALL. BCP-ALL, although less frequently than JMML, has been reported in some cases of NS. This association was first described in 1993 by Piombo et al. They presented an unrecognized NS case of a 30-month-old male who developed BCP-ALL and died of relapse after having achieved a complete remission. The first explanation of this association was related to a similar finding in the pathogenesis of the NF1 [32]. Yamamoto et al. explored the prevalence of mutations in the *PTPN11*, *RAS,* and *FLT3* genes in diagnostic samples from 95 Japanese children with ALL and identified missense mutations of *PTPN11* in exon 3 and 8 in six children with BCP-ALL and NS. The same mutation in 922 A>G (N308D) was detected in both NS and BCP-ALL [33]. Furthermore, it has been reported that patients with BCP-ALL carrying somatic mutations in *PTPN11* more frequently presented a hyperdiploid (HeH) karyotype than did those without *PTPN11* mutations [34]. A possible explanation for this finding could be that hyperdiploid clones may show the amplification mutant *PTPN11* allele but not the normal *PTPN11* allele, and this might over-activate the SHP-2 protein to a level promoting leukemogenesis. Moreover, the association of HeH ALL and NS is underestimated, based on our retrospective evaluation: among 302 cases of ALL, we found 3 cases of HeH and NS. For this reason, we hypothesized that NS is the second most common genetic disease predisposed to the development of ALL, after Down syndrome. NS has also been associated with the simultaneous occurrence of ALL and JMML in the same patient, as reported by Pauli et al. They presented a girl with NS harboring a *PTPN11* germline mutation c.417 G>C (p.E139D) who developed BCP-ALL at 16 months of age; during remission, at 4 years of age, JMML was diagnosed. A germline mutation in a heterozygous state was detected through the molecular genetic analysis of the lymphoblasts performed at the onset of ALL, while in the myelomonocytic blasts associated with JMML, the mutation p.E139D was identified to be in a homozygous state due to a uniparental disomy. Based on these findings, the pathogenesis of ALL and JMML in this patient is related to different mechanisms due to the occurrence of somatically acquired secondary chromosomal abnormalities [35]. While NS is frequently associated with BCP-ALL, T-lineage ALL is less common. One of the few cited cases was described by Kaya et al. A 9-year-old boy was diagnosed with medium-risk T-ALL while receiving growth hormone (GH) therapy for the treatment of short stature due to NS. At the end of treatment, he maintained the complete remission [36]. Germline mutations in the *PTPN11* gene cause NS, as well as other syndromes such as LEOPARD syndrome (LS), an uncommon congenital disorder characterized by multiple lentigines, cardiac involvement, facial dysmorphism, retardation of growth, and deafness. The difference between the two syndromes is related to the exons in which the mutation is located: the two most frequent mutations found in LS are the heterozygous missense mutations Y279C in exon 7 and T468M in exon 12. Instead, NS mutations are clustered in exons 3 and 8, respectively. Laux et al. presented the case of an 8-year-old girl with LS who developed B-ALL (Table 4) [37]. So far, whether and how this type of mutation can affect the risk of developing hematologic malignancies still needs to be clarified. The *PTPN11* gene is mainly involved in the development of NS, but is not the only gene involved; in recent years, novel genes have been described relating to NS, including *RRAS*, *RASA2*, *LZTR1*, *SOS2*, *A2ML1*, and *PPP1CB*. Chinton et al. studied 14 patients with NS and the germline variants in the *LZTR1* gene. Among them, 50% presented heart defects and neurodevelopmental delay or learning disabilities, 21% displayed short stature, and one patient developed ALL, showing that leukemia could also be present with other mutations occurring in NS [38].

### 5.4. Noonan Syndrome (NS) and Other Malignancies

The association between NS and hematologic malignancies is relevant, but this relationship could also extend to solid tumors. The majority of solid cancers occurring in NS patients includes rhabdomyosarcoma, neuroblastoma, and glioma [4]. Some of these tumors carried uncommon mutations or presented with unusual diagnoses (Table 5). Cianci et al. reported the association between Burkitt lymphoma and NS due to an *RAF1* gene mutation [39]. The *RAF1* gene mutations show low incidence, accounting for 5–10% of NS cases, with many patients presenting with hypertrophic cardiomyopathy as a specific cardiologic feature. The reported patient was diagnosed with NS at 5 months of age, and when he was 7 years old, he manifested a t(8;14) positive Burkitt lymphoma. He achieved complete remission, without any side effects related to the chemotherapy [39]. Also in the field of lymphoma, Avery et al. described a patient with Noonan-like syndrome with loose anagen hair (NS/LAH), caused by a germline mutation in *SHOC2*, who presented with cutaneous T-cell lymphoma [40]. This mutation is very rare in NS and account for 1,5% of NS cases [40]. It is not only associated with NS but also with this uncommon disorder, presenting with additionally ectodermal abnormalities, e.g., thin loose anagen hair, cutaneous hyperpigmentation, palmar/plantar wrinkling, hyper-elastic skin, and atopic dermatitis. Indeed, the *SHOC2* gene modulates the RAS/MAPK signaling pathway but also the proliferation and differentiation of epithelial stem cells in the skin and hair follicles, thus suggesting the association with cutaneous lymphoma as well [40]. Another rare association is a link with colorectal cancer. Prasad et al. reported the case of girl diagnosed with NS associated with a germline mutation *SOS1* gene, who, at 14 years of age, presented with colon adenocarcinoma, without any association with other hereditary cancer syndromes [41]. Tumor analysis revealed three mutations: one germline (*SOS1* pathogenic for NS) and two somatic (a *TP53* missense mutation and *NCOR1* nonsense mutations). A possible explanation of this case is that the Ras signaling pathway has been shown to play an important role in the pathogenesis of adult colorectal cancer (CRC), and some of the same somatically mutated genes causing adult CRC could be mutated in the germline of many cancer-predisposition syndromes. The RAS/MAPK pathways are also implicated in the development of sporadic low-grade glial tumors comprising disembryoplastic neuroepithelial tumors (DNETs). DNETs typically present as rare, solitary, benign, WHO grade 1, cortical tumors, commonly detected in the temporal lobes and considered to be a frequent cause of intractable epilepsy [42]. They could be part of the tumor spectrum associated with *PTPN11*-driven NS, as reported by Siegfried et al. [43]. Furthermore, McWilliams et al. presented the case of an 8-year-old boy with NS, confirmed by identification of a *PTPN11* mutation, who presented with DNET while receiving growth hormone (GH) treatment due to his short stature. Even though a clear relationship between GH therapy and brain tumor onset has not been demonstrated, it is recommended that physicians should be aware of the possibility of increased neoplasia risk and that they should make judicious use of GH therapy for patients with tumor-predisposition syndromes such as NS [44]. As reported above, NS is commonly associated with glioma as CNS tumors. Lodi et al. described a 13-year-old girl with NS related to a *PTPN11* mutation who developed a glioneuronal neoplasm of the left temporal lobe. Molecular characterization of this tumor revealed high levels of phosphorylated mTOR (pMTOR); therefore, a therapy based on an mTOR inhibitor (everolimus) was administered. The treatment was well tolerated, leading to a stabilization of the tumor, which was surgical removed [45]. Within the gliomas associated with NS, the optic pathway tumors are rare, but are frequently manifested in about 15% of patients with NF-1 below the age of 6 years. Although this event is unlikely, Sair et al. described the case of a 14-year-old boy with NS and *PTPN11* mutation who was diagnosed with optic nerve pilomyxoid astrocytoma [46]. Despite being rare, sub-ependymoma associated with NS has also been reported. Boonyawat et al. presented the case of an 11-year-old patient with NS and a *PTPN11* mutation who was diagnosed with subependymoma in the fourth ventricle [47]. Although there is no certain relationship between the specific *PTPN11* mutation and the incidence of cancer, almost 15% of brain tumors in *PTPN11* mutation-associated NS were associated with the heterozygous form of the p.Asn308Asp mutation. Therefore, attention should be focused on investigating CNS tumors in patients with NS bearing a *PTPN11* mutation.

*PTPN11* mutations and the RAS pathway may also play a role in the pathogenesis of rhabdomyosarcoma (RMS) [48]. Indeed, Jongmans et al. reported an NS patient with a de novo germline *SOS1* mutation (identified in about 13% of NS cases) who developed an embryonal RMS at four years of age. The heterozygous germline mutation was homozygously present in the embryonal RMS of the child. The authors hypothesized that there could be an association between this germline mutation of the *SOS1* gene and the tumor development. In order to prove this, they screened the DNA isolated from 20 cases with sporadic embryonal RMS for somatic mutations. Currently, no pathogenic mutations have been detected, suggesting that *SOS1* does not play an important role in the onset of embryonal RMS outside the context of NS, identifying this as an uncommon event [49].

**Table 5:** Cases of NS associated with other malignancies, as cited in the literature.

References	Cases (Age—Gender)	Type of Tumor	Genetic Aberration
Jongmans et al. [49]	4-year-old male	Embryonal RMS	c.2183 A>T (p.Lys728Ile) in *SOS1*
Cianci et al. [39]	7-year-old male	Burkitt lymphoma	c.776 C>T (p.Ser259Phe) in *RAF1*
Nair et al. [46]	14-year-old male	Astrocytoma	c.417G>C in exon 4 of *PTPN11*
McWilliams et al. [44]	8-year-old male	DNET	p.Glu139Asp in *PTPN11*
Prasad et al. [41]	14-year-old female	Colorectal cancer	c.1310 T>C (p. Ile437Thr) in SOS1
Boonyawat et al. [47]	11-year-old female	Subependymoma	c.922 A>G(p.Asn308Asp) in *PTPN11*
Lodi et al. [45]	13-year-old female	Glioma	c.922 A>G, p.Asn308Asp in *PTPN11*
Avery et al. [40]	25-year-old female	T-cell lymphoma	c.4 A>G p.S2G in *SHOC2*

## 6. Recommendations and Conclusions

Since NS is often diagnosed later in life, each NS patient with hematological malignancy or solid tumor requires an individualized multidisciplinary management approach owing a different prognosis based on individual symptoms and disease severity. Despite the presence of a hematological disease or a solid tumor, these patients need to be followed throughout their lifetimes in order to understand the impact of the main genetic aberration on the onset of secondary tumors or other debilitating diseases [13]. It is well known that guidelines are available for NS management [1,50], inducing the involved health care providers to increase beneficial lifelong patient outcomes. Conversely, the outcome for children and adolescents with NS and hematological malignancies or solid tumors is currently unknown because of the lack of early identification and the rare incidence among pediatric cases enrolled in current protocols; thus, a long follow-up period could yield this invaluable information. A prospective collaborative worldwide study should be proposed and performed.

At the end of this review, based on our experience and considerations, NS is confirmed to be a cancer-prone or cancer-predisposing syndrome. It could be strongly associated with hematological and solid malignancies. The germline mutations causing this syndrome often occurred as somatic mutations in cancers related to the NS, such as ALL or JMML. Our paradigmatic case (Case 1), apart from reporting the ALL vs. JMML sequence closely related to the *PTPN11* germline alteration, showed the rarely described presentation of a TMD at birth, as is common in Down syndrome. Furthermore, the NS diagnosis was made after the ALL onset, and in our center, an incidence of 1% of NS cases with a secondary molecular diagnosis was retrospectively demonstrated. Considering the results of our literature overview, our case presents as a unique example, reminding healthcare providers to consider this syndrome as a possible cause behind some hematological malignancies associated with a specific phenotype. The opposite should also be considered, e.g., healthcare providers should also suspect the incidence of a hematological disease in a patient with NS in order to reach an early diagnosis. Strikingly, our review strongly suggests that Noonan syndrome is the second most common cancer-predisposing syndrome, after Down syndrome, in children with ALL, with the literature confirming that this relationship is currently underestimated.

## Figures and Tables

**Table 1:** Summary of chronological events occurring in **Case 1 (UPN 1045685)** over her lifetime.

Age	Type of Alteration	Management
At birth	Neoformation diagnosed, with juvenile xantogranuloma-like Langherans histiocytosis	No treatment
First week	Respiratory distress and sepsis caused by Pseudomonas aeruginosa	Ventilatory support and intravenous antibiotic therapy
First Month	Leukocytosis and splenomegaly diagnosed as transient myeloproliferative disorder (TMD)	Spontaneous recovery
Second Year	B-cell precursor acute lymphoblastic leukemia (BCP-ALL)	AIEOP-BFM ALL 2009 protocol
Seventh Year	Genetic characterization of Noonan syndrome by identification of germline *PTPN11* mutation	No treatment
Tenth Year	Juvenile myelomonocytic leukemia (JMML)	6-mercaptopurine (final dose 50 mg/die)
Eleventh Year	Treatment failure characterized by hyperleukocytosis associated with thrombocytopenia and splenomegaly	Haploidentical stem cell transplantation (father as donor)

**Table 2:** Cases of NS associated with transient myeloproliferative disease (TMD), as cited in the literature.

References	Case (Gender and Age)	Type of Clinical Aberration	Genetic Aberration
Ferraris et al. [22]	Male—2 months old	Transient Abnormal Myelopoiesis	Not identified
Kratz et al. [4]	Male—10 months old	Leukopenia and TMD	c.69A>T (p. Lys23Asn) in *RIT1*
Bastida et al. [24]	Male—3 months old	TMD	(F285S) in exon 8 of the *PTPN11* gene
Bastida et al. [24]	Male—22 days old	TMD	(D61G) in exon 3 of the *PTPN11* gene
O’Halloran et al. [25]	At birth	Transient JMML and monosomy 7	Ser502Leu in *PTPN11*

**Table 3:** Cases of NS associated with JMML, as cited in the literature.

Reference	Case	Type of Hematological Malignancy	Genetic Aberration
Choong et al. [28]	At birth	JMML	Not specified
Cheong, J.L. et al. [29]	At birth	JMML	*PTPN*11 mutation C-T substitution at 218
Ortiz et al. [30]	16 years old	JMML	*KRAS*2 missense mutation at codon 13 (Gly-Cys)
Strullu, M. et al. [26]	20 cases	JMML	See reference [26]

**Table 4:** Cases of NS associated with ALL, as cited in the literature.

References	Cases	Type of Hematological Malignancy	Genetic Aberration
Piombo et al. [32]	30-month-old	BCP-ALL	*PTPN11* mutation not specified
Yamamoto et al. [33]	6 out of 95 children	BCP-ALL	922A>G (N308D) in *PTPN11*
Laux et al. [37]	8-year-old	BCP-ALL ^	c.836 A>G, in exon 7 in *PTPN11*
Pauli et al. [35]	4-year-old	BCP-ALL and JMML	c.417 G>C (p.E139D) in *PTPN11*
Sakamoto et al. [34]	6-year-old	BCP-ALL	c.922 A>G; (p. N308D) in *PTPN11*
Chinton et al. [38]	3-year-old	BCP-ALL	c.742 G>A (p. Gly248Arg) in *LZTR1*
Kaya et al. [36]	9-year-old	T-ALL	1502 G>A (p. Arg501Lys) in *PTPN11*

^ = in a case with LEOPARD syndrome.

## Data Availability

The original contributions presented in the study are included in the article, further inquiries can be directed to the corresponding author.
